# Pain management in chronic limb-threatening ischaemia: a multi-centre cross-sectional study

**DOI:** 10.3389/fmed.2026.1876171

**Published:** 2026-09-16

**Authors:** Huiping Mi, Kai Cao, Weiliang Yang, Xin Yao, Weifeng Wang, Jiaqing Luo, Junwen Peng, Zhenjie Liu

**Affiliations:** 1 Department of Nursing, The Second Affiliated Hospital, Zhejiang University School of Medicine, Hangzhou, China; 2 Department of Vascular Surgery, The Second Affiliated Hospital, Zhejiang University School of Medicine, Hangzhou, Zhejiang, China; 3 Department of Vascular Surgery, Suichang People’s Hospital, Suichang, China; 4 Department of General Surgery, Quzhou Second Hospital, Quzhou, China; 5 Department of Vascular Surgery, Xinchang People’s Hospital, Xinchang, China; 6 Department of Vascular Surgery, Changxing People’s Hospital, Changxing, China; 7 Department of General Surgery, Jiande People’s Hospital, Hangzhou, Zhejiang, China

**Keywords:** analgesia, brief pain inventory, China, chronic limb-threatening ischaemia, clinical guidelines, opioid access, pain management, peripheral arterial disease

## Abstract

**Background:**

Chronic limb-threatening ischaemia (CLTI), the most advanced form of peripheral arterial disease, is associated with severe ischaemic pain that profoundly affects quality of life. Despite this, pain management in CLTI has received limited research attention, particularly in China. We carried out a multi-centre cross-sectional study to assess pain severity, functional impact, and analgesic prescribing among hospitalised CLTI patients in Zhejiang Province.

**Methods:**

Between January 2024 and December 2024, we recruited 236 consecutive inpatients with CLTI from six hospitals: one tertiary centre and five county-level hospitals. Eligible patients were aged >=18 years, had a primary diagnosis of CLTI (Fontaine stage III-IV or Rutherford 4–6), were experiencing pain, and could provide a reliable self-assessment. Pain was evaluated using the Brief Pain Inventory. Prescribing data were extracted from electronic medical records. The primary outcome was the proportion with moderate-to-severe pain (NRS >=5/10).

**Results:**

The median age was 72 years and 66.1% were male. A large majority (88.6%) reported moderate-to-severe pain. Median scores were: current pain 6/10, worst pain 8/10, average pain 6/10. Pain interfered most with walking ability (median 8/10), sleep (7/10), and general activity (7/10). Analgesic use was limited: paracetamol 74.2%, weak opioids 34.7%, morphine 30.5%, oxycodone 22.5%. Eight patients (3.4%) received no analgesic therapy. Among those with severe pain (>=7/10), 18.8% were not prescribed any strong opioid. Overall, 54.7% reported inadequate analgesia (<=50% pain relief). Significant urban-rural disparities were observed in strong opioid prescribing.

**Conclusion:**

Pain management among hospitalised CLTI patients in this Chinese cohort is frequently suboptimal. The high prevalence of uncontrolled pain, low utilisation of strong opioids, and marked functional impairment point to an opportunity for quality improvement for improved practice. Given the single-time-point nature of this assessment, these findings highlight a critical gap in initial symptom control. Development of China-specific clinical guidelines, wider adoption of nursing-led pain assessment, and better access to analgesics in county hospitals should be prioritised. Future clinical trials of multimodal strategies for ischaemic pain are also warranted.

## 1 Introduction

Chronic limb-threatening ischaemia (CLTI) is the most severe manifestation of peripheral arterial disease (PAD), defined by ischaemic rest pain lasting at least two weeks and/or tissue loss attributable to arterial insufficiency (1, 2). In China, an estimated 5.6 to 7.8 million people live with CLTI, and the burden is expected to grow as the population ages and diabetes prevalence rises (3–7, 25). The prognosis is poor: within one year of diagnosis, approximately 30% of patients require major amputation and 25% die (1, 8, 9, 36, 37).

Pain is a central feature of CLTI. It arises from multiple mechanisms – ischaemia and inflammation produce nociceptive pain, chronic ischaemia leads to neuropathic changes, and in patients with diabetes there may be concomitant painful diabetic neuropathy (10–12). The pain is typically persistent, worse at night, and position-dependent (aggravated by limb elevation, partially eased by dependency). Sleep disruption is common, and the overall impact on daily life is substantial (13).

Given the severity of CLTI-associated pain, one would expect aggressive analgesic management. Yet available evidence suggests this is often not the case. The Global Vascular Guidelines note that pain management is frequently inadequate in this population and call for a multimodal approach (1). Current consensus documents acknowledge the lack of robust evidence to guide analgesic selection in vascular disease (14–16, 26). In short, clinicians have limited guidance, and patients may suffer unnecessarily.

In China, several factors may compound the problem. PAD awareness and revascularisation rates are low (3, 5). Regulatory restrictions make strong opioids difficult to prescribe, especially outside major cities. Diabetes-related CLTI is common. The role of traditional Chinese medicine in CLTI pain management is not well defined. Against this background, we designed a cross-sectional study to describe pain severity, functional interference, and prescribing patterns among hospitalised CLTI patients across tertiary and county-level hospitals in Zhejiang Province.

## 2 Materials and methods

### 2.1 Study design and setting

This was a cross-sectional observational study conducted from January 2024 to December 2024 at six hospitals in Zhejiang Province, eastern China: The Second Affiliated Hospital of Zhejiang University School of Medicine (tertiary centre, Hangzhou) and five county-level public hospitals (Jiande, Suichang, Quzhou Second, Xinchang, and Changxing People's Hospitals). The county hospitals serve predominantly rural and semi-urban populations. Reporting follows the STROBE guidelines (17).

### 2.2 Participants

Consecutive inpatients aged >=18 years with a primary diagnosis of CLTI were screened. CLTI was defined according to the Global Vascular Guidelines (1, 27) as chronic ischaemic rest pain (>2 weeks) and/or tissue loss (arterial ulceration or gangrene) with objectively confirmed PAD (ABI <0.90, toe-brachial index <0.70, or imaging evidence of significant stenosis/occlusion). Inclusion criteria were: age >=18; primary diagnosis of CLTI (Fontaine stage III-IV or Rutherford category 4–6) (28, 29); currently experiencing CLTI-related pain; and able to provide a reliable self-reported pain assessment. Exclusion criteria were: cognitive impairment, severe psychiatric illness, or profound frailty precluding reliable assessment; pain solely attributable to non-ischaemic causes; or enrolment in an interventional trial affecting analgesic prescribing.

### 2.3 Data collection

A structured case report form was developed for this study. Demographic data, clinical characteristics (Fontaine stage, Rutherford category, disease duration, ABI, comorbidities, smoking status, renal function), pain assessment, prescribing records, and efficacy outcomes were recorded.

Pain was assessed using the Brief Pain Inventory short form (BPI-SF), a validated instrument with established reliability and responsiveness in chronic pain populations (18–22). Part A (pain severity) asks patients to rate current, worst, least, and average pain over the past 24 h on a 0–10 numeric rating scale (0 = no pain, 10 = worst possible pain). Part B (pain interference) asks patients to rate how much pain has interfered with seven aspects of daily life on the same 0–10 scale (0 = does not interfere, 10 = completely interferes) (32). The Mandarin Chinese version of the BPI-SF has been validated in Chinese populations (19, 33, 34).

Analgesic prescribing data were extracted from electronic medical records by trained research nurses. Medications were categorised as: paracetamol; weak opioids (codeine, tramadol); strong opioids (morphine, oxycodone, fentanyl); gabapentinoids; NSAIDs; antidepressants; patient-controlled analgesia; local anaesthetics; traditional Chinese medicine modalities; and other analgesics.

### 2.4 Outcomes

The primary outcome was the proportion of patients with moderate-to-severe pain, defined as a current pain NRS >=5/10. Secondary outcomes were: distribution of pain severity scores; pain interference scores across seven BPI domains; analgesic prescribing patterns; proportion reporting inadequate analgesia (<=50% pain relief); proportion with severe pain (>=7/10) not prescribed any strong opioid; and proportion receiving no analgesic therapy.

### 2.5 Statistical analysis

Data were analysed using SPSS version 26.0 (IBM Corp.). Categorical variables are expressed as frequencies and percentages. Continuous variables are reported as medians with interquartile ranges (IQR) because pain scores were not normally distributed. Between-group comparisons of pain scores used the Wilcoxon rank-sum test. Categorical comparisons used the chi-squared or Fisher's exact test. Two-sided *p* < 0.05 was considered significant. Missing data were not imputed.

### 2.6 Ethics

The study was approved by the ethics committee of The Second Affiliated Hospital of Zhejiang University School of Medicine (approval number 2023-Ethics-0892) and by the ethics committees of all participating hospitals. All participants or their legally authorised representatives provided written informed consent. The study was conducted in accordance with the Declaration of Helsinki.

## 3 Results

### 3.1 Patient characteristics

A total of 236 patients were enrolled. The tertiary centre contributed 142 (60.2%) and the five county hospitals 94 (39.8%). Demographic and clinical characteristics are shown in Table 1. Median age was 72 years (IQR 65–77) and 66.1% were male.

**Table 1:** Demographic and clinical characteristics of 236 hospitalised patients with chronic limb-threatening ischaemia.

Characteristic	Value	Characteristic	Value
Age, median (IQR), years	72 (65–77)	Laterality	
Male	156 (66.1)	Left	76 (32.2)
Female	80 (33.9)	Right	66 (28.0)
BMI, median (IQR), kg/m^2^	24.5 (21.8–27.2)	Bilateral	94 (39.8)
Fontaine stage		ABI, median (IQR)	
III	82 (34.7)	Left	0.51 (0.38–0.68)
IV	154 (65.3)	Right	0.50 (0.36–0.66)
Rutherford category		Comorbidities	
4 (Rest pain)	100 (42.4)	Hypertension	215 (91.1)
5 (Minor tissue loss)	89 (37.7)	Diabetes mellitus	123 (52.1)
6 (Major tissue loss)	47 (19.9)	Dyslipidaemia	98 (41.5)
Disease duration, median (IQR), months	8 (3–16)	CKD	82 (34.7)
Diagnosis basis		CAD	67 (28.4)
Rest pain >2 weeks	90 (38.1)	Stroke/TIA history	36 (15.3)
Tissue loss/ulceration	75 (31.8)	Smoking (current/former)	91 (38.6)
Both	71 (30.1)	HbA1c (diabetics), median (IQR), %	8.2 (6.9–9.8)
eGFR, median (IQR), mL/min/1.73m^2^	58 (42–76)		
Revascularisation status		Amputation status	
No	139 (58.9)	No	171 (72.5)
Previous	44 (18.6)	Previous	31 (13.1)
Planned	52 (22.0)	Planned	36 (15.3)
Not candidate	43 (18.2)		
Admission reason		Centre	
Rest pain	89 (37.7)	Tertiary centre	142 (60.2)
Tissue loss/infection	75 (31.8)	County hospitals	94 (39.8)
Vascular intervention	52 (22.0)		
Other	20 (8.5)		

Disease was advanced: 65.3% were Fontaine stage IV; Rutherford categories were 4 in 42.4%, 5 in 37.7%, and 6 in 19.9%. Median disease duration was 8 months (IQR 3–16). Bilateral lower limb involvement was present in 39.8%. Median ABI was 0.51 on the left (IQR 0.38–0.68) and 0.50 on the right (IQR 0.36–0.66).

Comorbidities were common: hypertension 91.1%, diabetes mellitus 52.1%, dyslipidaemia 41.5%, chronic kidney disease 34.7%, coronary artery disease 28.4%, and prior stroke or transient ischaemic attack 15.3%. Regarding treatment status, 18.6% had undergone prior revascularisation, 22.0% had planned revascularisation, and 18.2% were deemed unsuitable for revascularisation. The main reasons for hospitalisation were ischaemic rest pain (37.7%), tissue loss or infection (31.8%), and scheduled vascular intervention (22.0%).

### 3.2 Pain severity and functional impact

Pain severity and functional interference are shown in Table 2. Median current pain was 6/10 (IQR 4–8), worst pain 8/10 (IQR 6–10), least pain 3/10 (IQR 1–5), and average pain 6/10 (IQR 4–8). Overall, 88.6% of patients (209/236) had moderate-to-severe pain (>=5/10) and 58.5% (138/236) had severe pain (>=7/10). Over half (52.1%) said their pain was worse than usual during the current hospital stay.

**Table 2:** Pain severity scores (brief pain inventory part A) and pain interference with daily function (brief pain inventory part B).

A. Pain Severity (BPI Part A)	Median (IQR)	n (%)
Current pain (0–10)	6 (4–8)	
Worst pain in 24 h (0–10)	8 (6–10)	
Least pain in 24 h (0–10)	3 (1–5)	
Average pain in 24 h (0–10)	6 (4–8)	
None (0)		4 (1.7)
Mild (1–3)		23 (9.7)
Moderate (4–6)		71 (30.1)
Severe (7–10)		138 (58.5)
Moderate-severe (>=5)		209 (88.6)
Pain worse than usual		123 (52.1)
B. Pain Interference (BPI Part B)	Median (IQR)	Severe (>=7), n (%)
General activity	7 (5–9)	142 (60.2)
Mood	6 (3–9)	98 (41.5)
Walking ability	8 (6–10)	167 (70.8)
Work	6 (3–9)	104 (44.1)
Relations with others	5 (2–8)	76 (32.2)
Sleep	7 (5–10)	145 (61.4)
Enjoyment of life	7 (5–9)	138 (58.5)

Pain had the greatest impact on walking ability (median interference score 8, IQR 6–10), followed by general activity (median 7, IQR 5–9), sleep (median 7, IQR 5–10), and enjoyment of life (median 7, IQR 5–9). Mood (median 6, IQR 3–9) and work (median 6, IQR 3–9) were moderately affected; interpersonal relations were least affected (median 5, IQR 2–8). Severe interference (>=7) was most common for walking ability (70.8%), sleep (61.4%), general activity (60.2%), and enjoyment of life (58.5%).

Subgroup comparisons showed no significant difference in current pain between the tertiary centre and county hospitals (median 6 vs. 6, *p* = 0.42), or between diabetic and non-diabetic patients (median 6 vs. 5, *p* = 0.18). However, the combination of advanced disease classification (65.3% Fontaine stage IV) and concomitant tissue loss or infection as a primary admission reason (31.8%) contributed to the high proportion of patients experiencing severe pain. Furthermore, our data indicates an inverse relationship between pain intensity and treatment efficacy: while 54.7% of the overall cohort reported inadequate analgesia, this failure rate escalated to 71.0% among those presenting with severe pain (NRS >= 7).

### 3.3 Analgesic prescribing and efficacy

Prescribing patterns and efficacy outcomes are shown in Table 3. Paracetamol was the most commonly used analgesic (74.2%, 175/236). Weak opioids were prescribed in 34.7% (82/236). Among strong opioids, morphine was used in 30.5% (72/236), oxycodone in 22.5% (53/236), and fentanyl in 7.2% (17/236). Gabapentinoids were prescribed in 20.8% (49/236), NSAIDs in 14.8% (35/236), and antidepressants in 14.4% (34/236). Traditional Chinese medicine analgesic modalities were documented in 15.7% (37/236).

**Table 3:** Analgesic prescribing patterns and efficacy outcomes among 236 hospitalised CLTI patients.

A. Analgesic Prescribing Patterns	n (%)
Paracetamol	175 (74.2)
Weak opioid (codeine/tramadol)	82 (34.7)
Morphine	72 (30.5)
Oxycodone	53 (22.5)
Fentanyl	17 (7.2)
Gabapentinoid	49 (20.8)
NSAID	35 (14.8)
Antidepressant (SSRI/SNRI)	27 (11.4)
Tricyclic antidepressant	7 (3.0)
Patient-controlled analgesia	7 (3.0)
Local anaesthetic	5 (2.1)
TCM/acupuncture	37 (15.7)
No analgesic therapy	8 (3.4)
Severe pain without strong opioid	26/138 (18.8)
B. Efficacy and Satisfaction	Value
Pain relief, median (IQR), %	40 (20–60)
Inadequate analgesia (<=50%)	129 (54.7)
Inadequate (among severe pain)	98/138 (71.0)
Sleep severely affected	108 (45.8)
Activity reduced due to pain	159 (67.4)
Satisfied/very satisfied	66 (28.0)
Dissatisfied/very dissatisfied	104 (44.1)

Of particular concern, 3.4% of patients (8/236) received no analgesic therapy during their hospital stay. Among patients with severe pain (>=7/10, *n* = 138), 18.8% (26/138) were not prescribed any strong opioid. There were significant differences in strong opioid prescribing between the tertiary centre and the county hospitals: morphine 34.5% vs. 23.4% (*p* = 0.03) and oxycodone 28.9% vs. 12.8% (*p* < 0.01).

The median reported pain relief from current analgesic therapy was 40% (IQR 20%–60%). Overall, 54.7% (129/236) reported inadequate analgesia (<=50% pain relief), rising to 71.0% among those with severe pain. Sleep was severely affected by pain in 45.8% of patients. Two-thirds (67.4%) had reduced their activity level because of pain. Satisfaction was poor: only 28.0% were satisfied or very satisfied, whereas 44.1% were dissatisfied or very dissatisfied.

## 4 Discussion

In this multi-centre cross-sectional study of hospitalised CLTI patients in Zhejiang Province, China, we found a high prevalence of moderate-to-severe pain (88.6%), substantial functional impairment, and low utilisation of strong opioids with significant urban-rural variation. Several points deserve emphasis.

### 4.1 The scale of uncontrolled pain

That nearly nine in ten hospitalised CLTI patients had pain rated as moderate-to-severe is a sobering finding. These are patients already admitted to hospital – one might expect their pain to be under better control. Yet the median worst pain score over the previous 24 h was 8/10, and more than half felt their pain was worse than usual during the admission. This suggests that hospitalisation, at least in its current form, does not reliably bring pain relief in this population.

The functional impact was equally concerning. Walking ability was most affected (median interference 8/10), which is especially problematic in CLTI because limited mobility exacerbates peripheral ischaemia and reduces the already narrow therapeutic window for revascularisation. Sleep disturbance (median 7/10) was also common, consistent with the well-described pattern of nocturnal rest pain in CLTI. Chronic sleep disruption has been linked to impaired wound healing, heightened pain sensitivity, and reduced quality of life (13, 31).

### 4.2 Why is analgesic prescribing so limited?

Several factors likely contribute. Because this is an inpatient cohort, it naturally selects for patients at the most severe end of the peripheral arterial disease spectrum. Intermittent claudication is appropriately managed in primary care; thus, by the time hospital admission is clinically indicated – typically due to intractable rest pain, tissue loss, or the need for intervention – the pain pathways are already deeply entrenched. This necessitates aggressive and immediate multimodal analgesic strategies upon admission.

Furthermore, opioid prescribing in China is governed by strict narcotic regulations. Physicians must undergo specialized training and institutional authorization to issue “Red Prescriptions” for strong opioids. The regulatory framework, which primarily focuses on cancer-related pain, imposes stringent dispensing limits for non-malignant chronic pain. This systemic barrier disproportionately affects county-level hospitals, where authorized prescribers and opioid quotas are often limited, contributing significantly to the observed under-treatment of severe ischaemic pain.

The relatively low use of gabapentinoids (20.8%) and NSAIDs (14.8%) is also noteworthy. Physicians may avoid gabapentinoids in elderly patients because of concerns about dizziness, falls, and cognitive effects (23). NSAID hesitancy is understandable given the comorbidity profile: 34.7% had CKD, 52.1% diabetes, 28.4% coronary artery disease – all conditions that increase the risk of NSAID-related complications.

### 4.3 The inadequacy of existing frameworks

The WHO analgesic ladder, originally developed for cancer pain, does not fit CLTI well (24). Ischaemic pain involves tissue hypoxia, inflammatory mediator release, and neuropathic changes that may not respond predictably to stepwise escalation of conventional analgesics. The fact that 54.7% of patients reported inadequate analgesia despite receiving one or more prescribed agents supports this view. The Global Vascular Guidelines (1) explicitly acknowledge the need for a multimodal approach, yet provide limited detail on how to construct it. Current consensus documents (14–16) similarly note the evidence gap. There is no CLTI-specific analgesic protocol, and clinicians are left to extrapolate from cancer pain or general chronic pain guidance.

### 4.4 What should be done?

At the clinical level, routine pain assessment with validated tools such as the BPI should become standard in vascular wards (35). Our nursing-led assessment model was feasible and well accepted. Expanding this approach through nurse training and electronic integration of BPI could improve detection and documentation of pain.

At the policy level, there is a case for reviewing how narcotic regulations affect legitimate clinical use. The current system appears to create unintended barriers for patients with severe, life-limiting ischaemic pain. Making specialised prescription permissions more accessible to vascular surgeons and pain specialists managing CLTI could help.

At the health system level, targeted support for county hospitals is needed. Telemedicine consultations with pain specialists, standardised analgesic protocols adapted to resource-limited settings, and continuing education on opioid prescribing for general physicians could reduce the urban-rural disparity we observed.

### 4.5 Directions for future research

This study was designed to describe the problem, not to test solutions. The next step should be clinical trials of multimodal analgesic strategies tailored to ischaemic pain mechanisms – including alpha-2 agonists, NMDA receptor antagonists, sodium channel blockers, and non-pharmacological options such as spinal cord stimulation. Implementation science research is also needed: once effective strategies are identified, how can they be embedded in routine vascular care? Health economic analysis should quantify the potential return on investment from better pain control in terms of reduced hospitalisations, improved wound healing, and delayed functional decline. Future studies should link pain outcomes with revascularisation strategy (30) and quality-of-life outcomes (31), and should identify no-option CLTI as a clinically distinct treatment context (38).

### 4.6 Strengths and limitations

Strengths include the multi-centre design spanning tertiary and county hospitals, the use of a validated pain instrument (BPI), the relatively large sample, and the combination of patient-reported and prescribing data. The county hospital focus enhances relevance to the broader Chinese CLTI population.

Limitations are those inherent to cross-sectional design: no causal inference, no capture of pain trajectories over time, and a single time-point assessment that may miss post-procedural fluctuations. Specifically, pain was assessed over the past 24 h during the patients' hospital stay. Because we did not track longitudinal data, we cannot determine whether physicians subsequently adjusted or escalated analgesic therapies in response to persistently high pain scores (e.g., NRS >6). Therefore, these findings represent a snapshot of concurrent pain and prescribing patterns rather than a dynamic evaluation of analgesic titration. EMR-based prescribing data may miss over-the-counter or traditional medicines not formally recorded. The study was confined to Zhejiang Province, so generalisability to other regions of China is uncertain. The predominance of patients from a single tertiary centre (60.2%) may have introduced selection bias.

## 5 Conclusion

This study documents suboptimal pain management among hospitalised CLTI patients in China. Nearly nine in ten experienced moderate-to-severe pain, more than half described their analgesia as inadequate, and a small but worrying minority received no analgesic therapy at all. Strong opioid use was low, with significant urban-rural disparities. Pain severely compromised walking ability, sleep, and daily activity.

Improved pain management in CLTI requires a concerted effort. Priorities should include the development of clinical practice guidelines specific to CLTI pain, wider adoption of nursing-led pain assessment, policy measures to improve analgesic access in under-resourced hospitals, and clinical trials of multimodal strategies for ischaemic pain. Without such efforts, symptom control in CLTI will remain a significant clinical challenge.

## Data Availability

The datasets generated and/or analysed during the current study are not publicly available because they contain potentially identifiable clinical information, but are available from the corresponding author on reasonable request, subject to institutional and ethical restrictions.
